# Anatomical axes of the proximal and distal halves of the femur in a normally aligned healthy population: implications for surgery

**DOI:** 10.1186/s13018-017-0710-0

**Published:** 2018-01-31

**Authors:** Hamidreza Yazdi, Ara Nazarian, John Y. Kwon, Mary G. Hochman, Reza Pakdaman, Poopak Hafezi, Morteza Ghahremani, Samad Joudi, Mohammad Ghorbanhoseini

**Affiliations:** 1 Department of Knee Surgery, Firoozgar Hospital, Neuromusculoskeletal Research Center, Iran University of Medical Sciences, District 6, Beh Afarin, Tehran, Iran; 2 Orthopaedic Surgery, Harvard Medical School - Nazarian Lab, Center for Advanced Orthopaedic Studies, BIDMC, 330 Brookline Ave., RN 115, Boston, MA 02215 USA; 3 Carl J. Shapiro Department of Orthopaedics, Orthopaedic Surgery, Harvard Medical School, BIDMC, 330 Brookline Avenue, Boston, MA 02215 USA; 4 Department of Radiology, BIDMC, Harvard Medical School, Section Chief Emeritus, Musculoskeletal Imaging and Intervention, Boston, USA; 5 Breast Imaging, Department of Radiology, Brigham and Women’s Hospital, Harvard Medical School, Boston, USA; 6 McLean Hospital, Harvard Medical School, Boston, MA USA; 7 Department of Orthopaedic Surgery, Firoozgar Hospital, Iran University of Medical Sciences, District 6, Beh Afarin, Tehran, Iran; 8 Department of knee surgery, Firoozgar Hospital, Iran University of Medical Sciences, District 6, Beh Afarin, Tehran, Iran

**Keywords:** Femur, Alignment view X-rays, Intramedullary nailing, Anatomical axis, TKA

## Abstract

**Background:**

The anatomical axis of the femur is crucial for determining the correct alignment in corrective osteotomies of the knee, total knee arthroplasty (TKA), and retrograde and antegrade femoral intramedullary nailing (IMN). The aim of this study was to propose the concept of different anatomical axes for the proximal and distal parts of the femur; compare these axes in normally aligned subjects and also to propose the clinical application of these axes.

**Methods:**

In this cross-sectional study, the horizontal distances between the anatomical axis of the proximal and distal halves of the femur and the center of the intercondylar notch were measured in 100 normally aligned femurs using standard full length alignment view X-rays.

**Results:**

The average age was 34.44 ± 11.14 years. The average distance from the proximal anatomical axis to the center of the intercondylar notch was 6.68 ± 5.23 mm. The proximal anatomical axis of femur passed lateral to the center of the intercondylar notch in 12 cases (12%), medial in 84 cases (84%) and exactly central in 4 cases (4%). The average distance from the distal anatomical axis to the center of the intercondylar notch was 3.63 ± 2.09 mm. The distal anatomical axis of the femur passed medially to the center of the intercondylar notch in 82 cases (82%) and exactly central in 18 cases (18%). There was a significant difference between the anatomical axis of the proximal and distal parts of the femur in reference to the center of intercondylar notch (*P* value < 0.05), supporting the hypothesis that anatomical axes of the proximal and distal halves of the femur are different in the coronal plane.

**Conclusions:**

While surgeons are aware that the anatomical axis of the distal part of the femur is different than the anatomical axis of the proximal part in patients with femoral deformities, we have shown that these axes are also different in the normally aligned healthy people due to the anatomy of the femur in coronal plane. Also the normal ranges provided here can be used as a reference for the alignment guide entry point in TKA and antegrade and retrograde intramedullary femoral nailing.

## Background

The femur has two axes, mechanical and anatomical [1, 2]. These axes play an important role in determining accurate bone alignment, especially in corrective osteotomies around the knee, total knee arthroplasties (TKA) and femoral fracture fixations [1, 2]. Anatomical restoration of femoral and tibial alignment are important to achieve optimal functional recovery [3]. For insertion of the femoral component in TKA, the classic alignment (neutral mechanical axis and a joint line perpendicular to the mechanical axis) or anatomic alignment should be recreated [4, 5]. Many studies have evaluated the proximal morphology of the femur in adults and have demonstrated differences among different populations and races [6, 7]. To define the mechanical axis of the femur, a line is drawn from the center of the femoral head to the anatomic center of the knee [8]. The center of the femoral head can be found easily by using the Mose circle [8]. However, determining the anatomical center of the knee can be more problematic. Five different points for defining the anatomical center of the knee have been described including the center of soft tissue shadow at the level of articular cartilage, center of the tibia, center of the femoral condyles in the plane of the deepest point of the intercondylar notch, center of the tip of the tibial spine, and the intercondylar notch center [3, 8, 9]. According to these studies, there are different techniques to outline the anatomical axis of the femur [3, 10]. Based on previous studies, the proper entry point for the femoral alignment rod in TKA is located several millimeters medial to the midline [2]. Considering the difference in anatomical shape of the femur in coronal plane [10], we propose that the anatomical axis of the proximal and distal halves of femur should be evaluated separately.

The aim of this study was to propose the concept of different anatomical axes in the proximal and distal parts of the femur; compare these axes in normally aligned people in reference to the distance from the intercondylar notch of the femur; and also propose the application of these axes and their normal ranges for surgeries such as total knee arthroplasties and for comminuted proximal and distal femoral fractures, which the anatomy and alignment of the femur are completely distorted.

## Methods

**Fig. 1:**
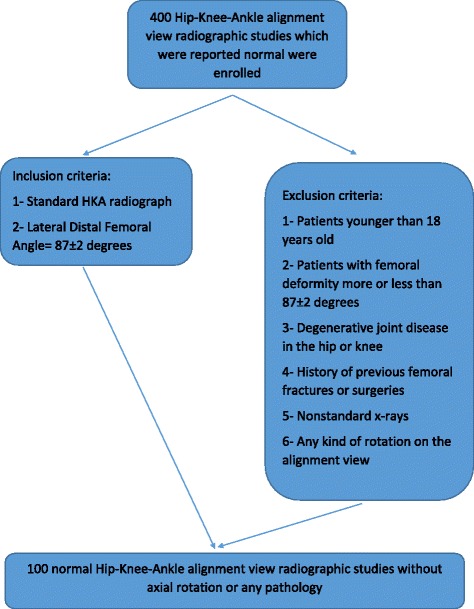
Subject selection process—original algorithm, not previously used in any article

Institutional review board approval was obtained for this study. In this cross-sectional study, we enrolled 400 cases referred to our university hospital from January of 2011 to December of 2014. All cases had digital anterior-posterior hip-knee-ankle (HKA) alignment view radiographic studies obtained for various reasons such as pre-recruit evaluation for military service or for suspected malalignments in physical exam. These X-rays were reported normal by a radiologist and were in compliance with the inclusion and exclusion criterion. Then we checked for any kind of rotation on the X-rays either caused by axial deformity or by lower limb rotation using anatomical landmarks and removed the cases that had axial rotation. The landmarks were the patella position, the proximal tibia-fibula overshadow (approximately 30%) and the appearance of the ankle joint [11]. At the end, we had 100 normal hip-knee-ankle alignment views (Fig. 1).

**Fig. 2:**
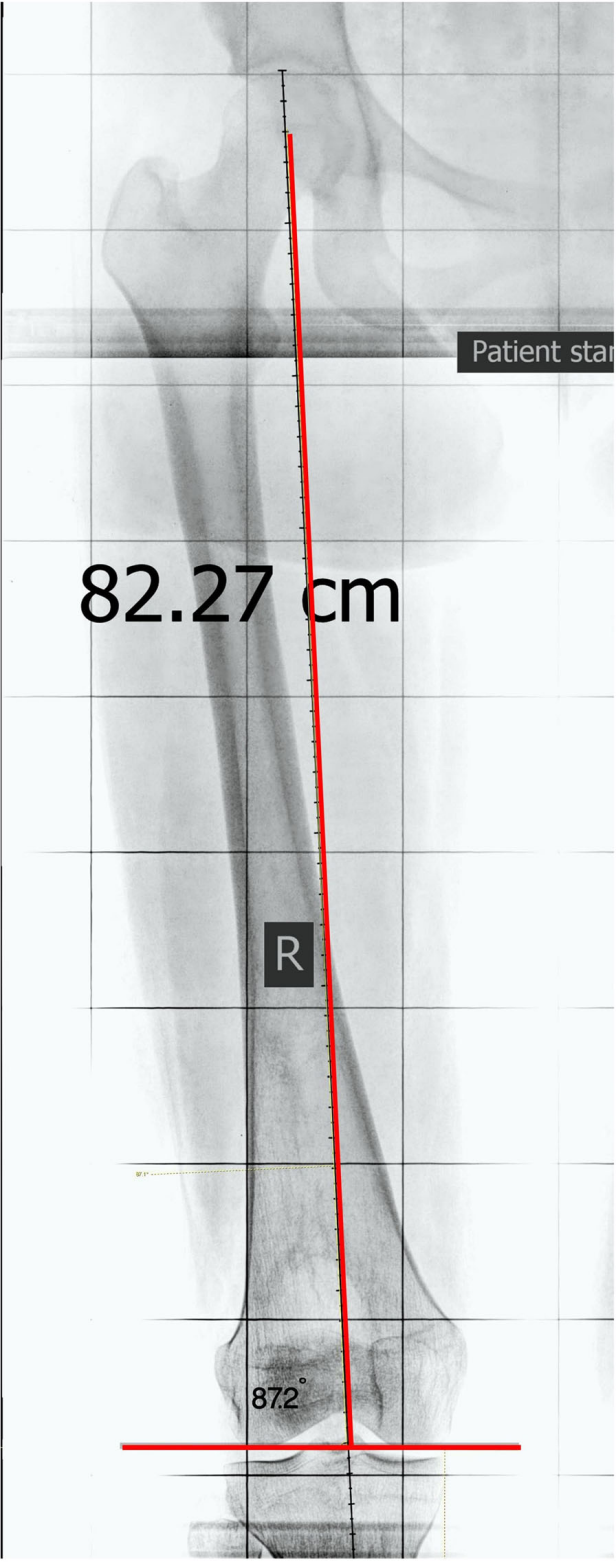
Normal aligned femur (lateral femora angle 87.2 degrees)—original image, not previously used in any article

The inclusion criterion was standard HKA radiograph with normal femoral alignment (lateral distal femoral angle = 87 ± 2 degrees) (Fig. 2) [12]. The exclusion criteria were subjects under 18 years old, subjects with femoral deformity (LDFA more or less than 87 ± 2 degrees), degenerative joint disease in the hip or the knee, history of previous femoral fractures or surgeries and/or nonstandard X-rays and any kind of rotation either caused by axial deformity or lower extremity rotation in the X-ray. All X-rays were re-evaluated by a staff radiologist and the primary investigator to meet the criteria for a standard HKA alignment view X-ray.

**Fig. 3:**
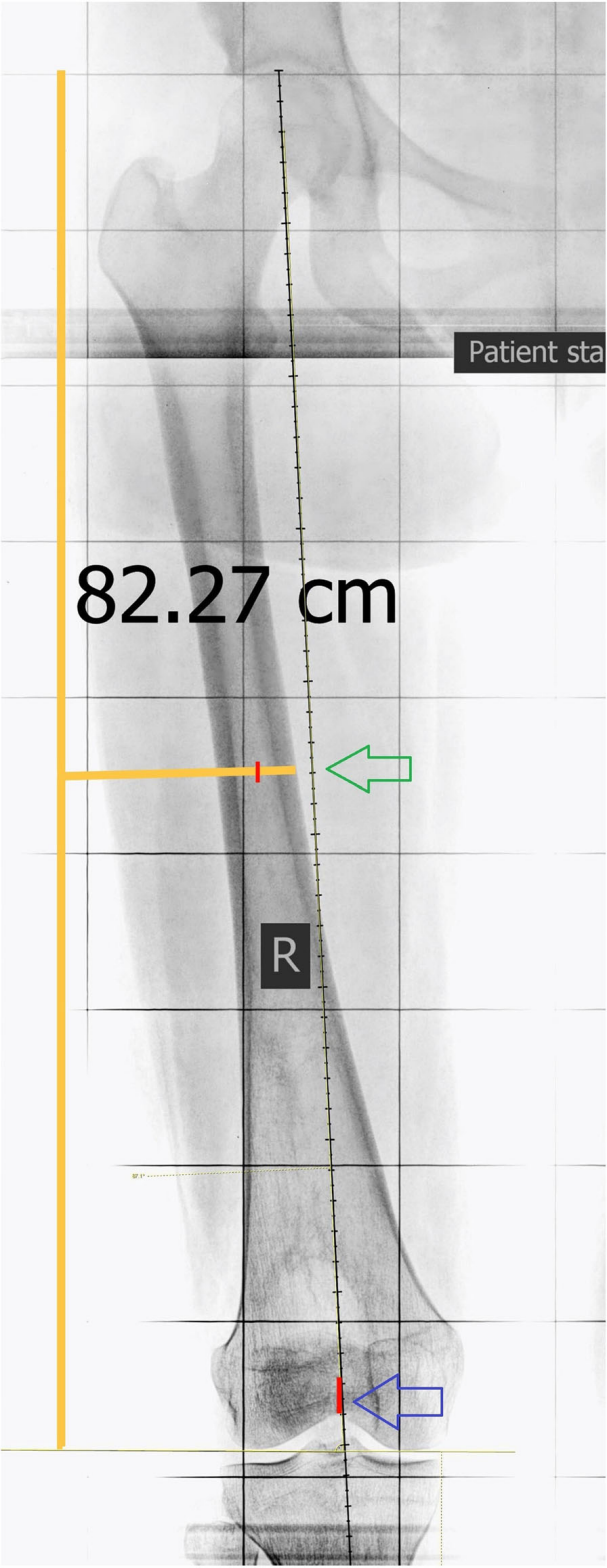
*Green arrow* points to the middle of the femur (midway between medial and lateral cortices), *blue arrow* points to the center of the intercondylar notch—original image, not previously used in any article

**Fig. 4:**
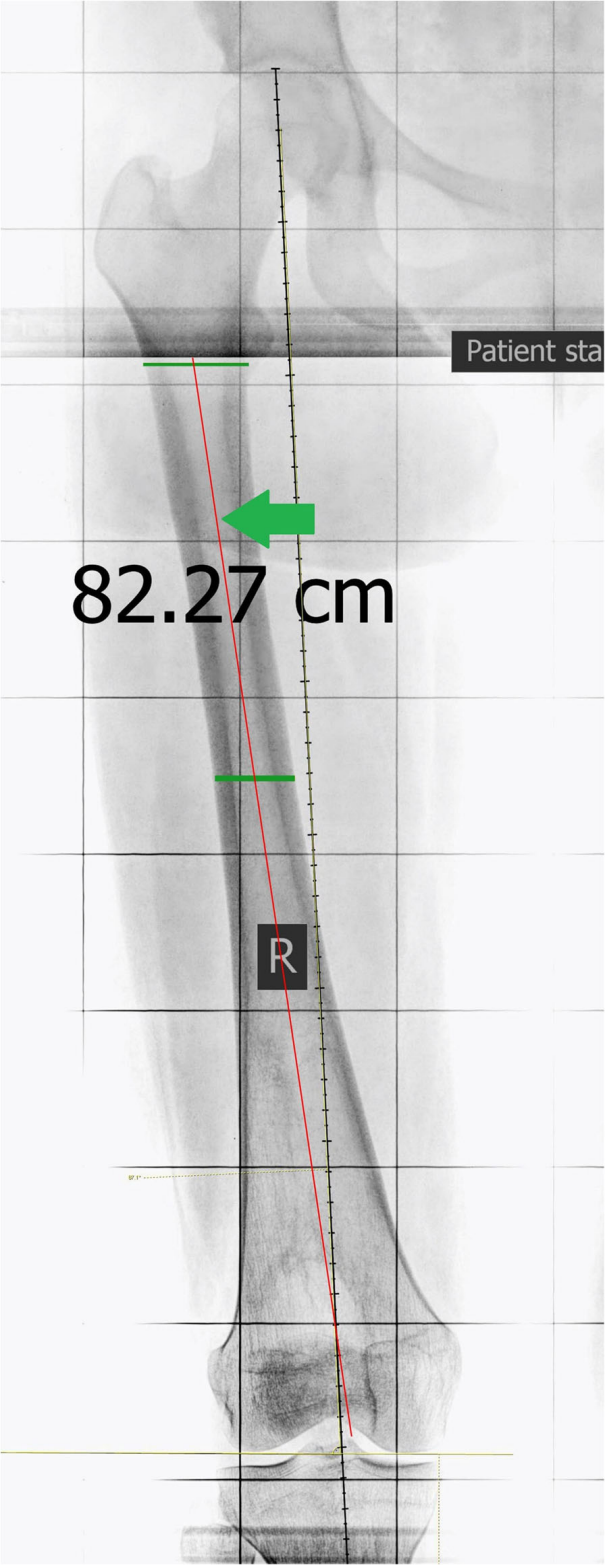
Anatomical axis of the proximal half of the femur (*green arrow*)—original image, not previously used in any article

**Fig. 5:**
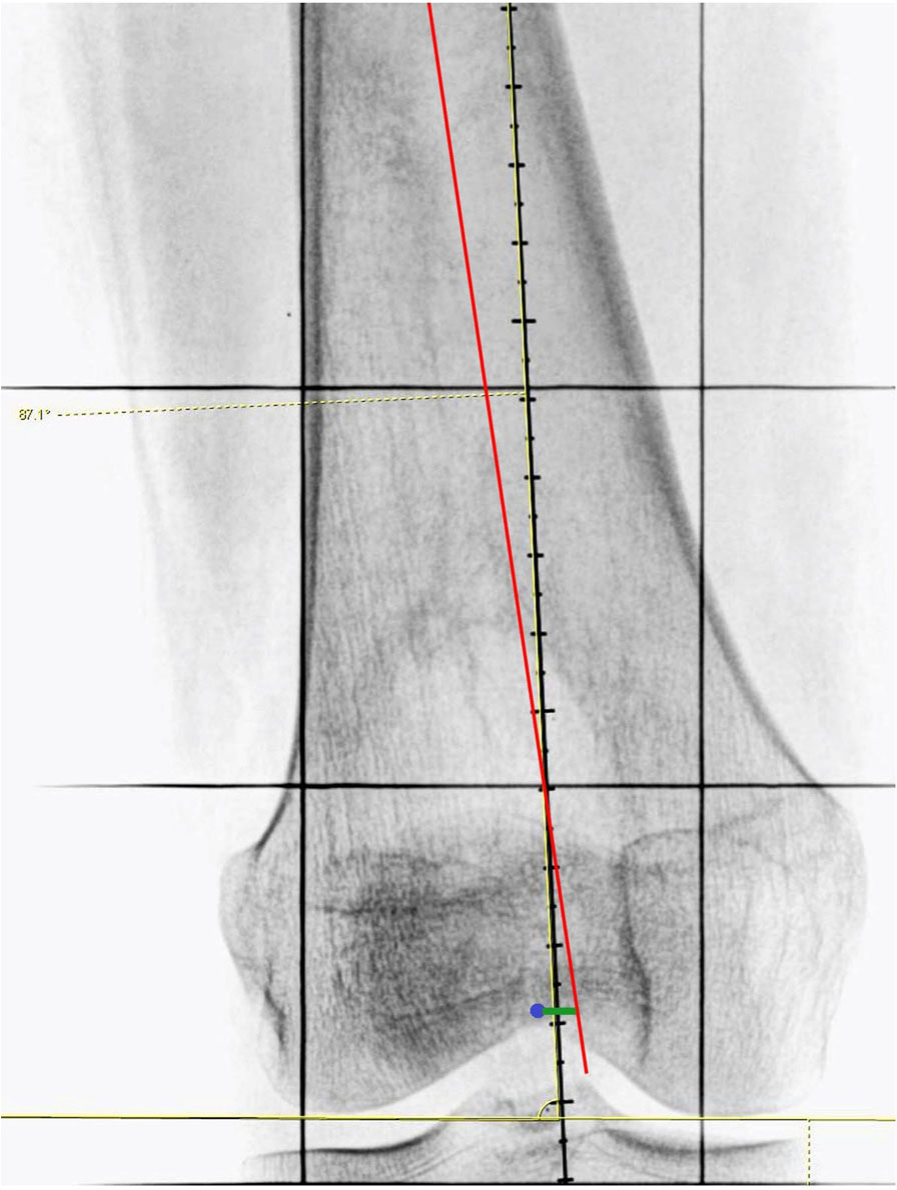
The distance (*green line*) between the projection of the anatomical axis of the proximal half of the femur (*red line*) and the center of intercondylar notch (*blue dot*)—original image, not previously used in any article

First the anatomical axis of the proximal femur was outlined according to the technique described by Morland et al. [3]. For this purpose, the midpoint of the medulla equidistant from the medial and lateral cortexes just below the lesser trochanter was determined. Then a line from this point to the midpoint of the medulla at mid shaft of femur (Fig. 3) was drawn and extended to cross the articular surface of femur (Fig. 4). The horizontal distance between this line and the center of the intercondylar notch was measured by Clear Canvas software (Synaptive Medical, Toronto, ON, Canada) in millimeters (Fig. 5). If the line passed medial to the center of the intercondylar notch, it was considered a positive measurement and vice versa.

**Fig. 6:**
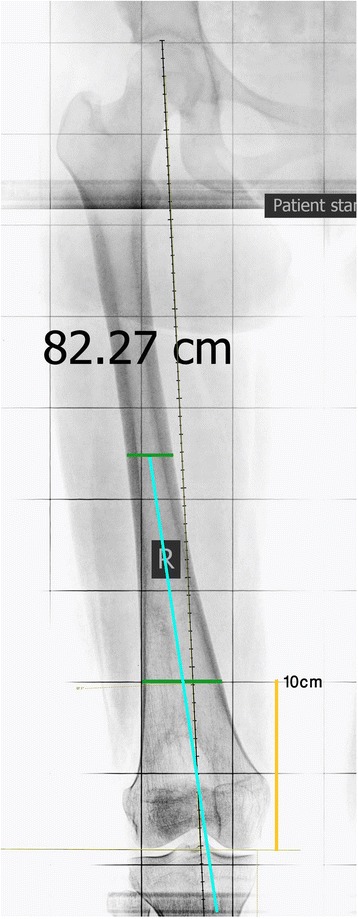
The anatomical axis of the distal half of the femur (*blue line*) according to the technique described by Morland et al.—original image, not previously used in any article

**Fig. 7:**
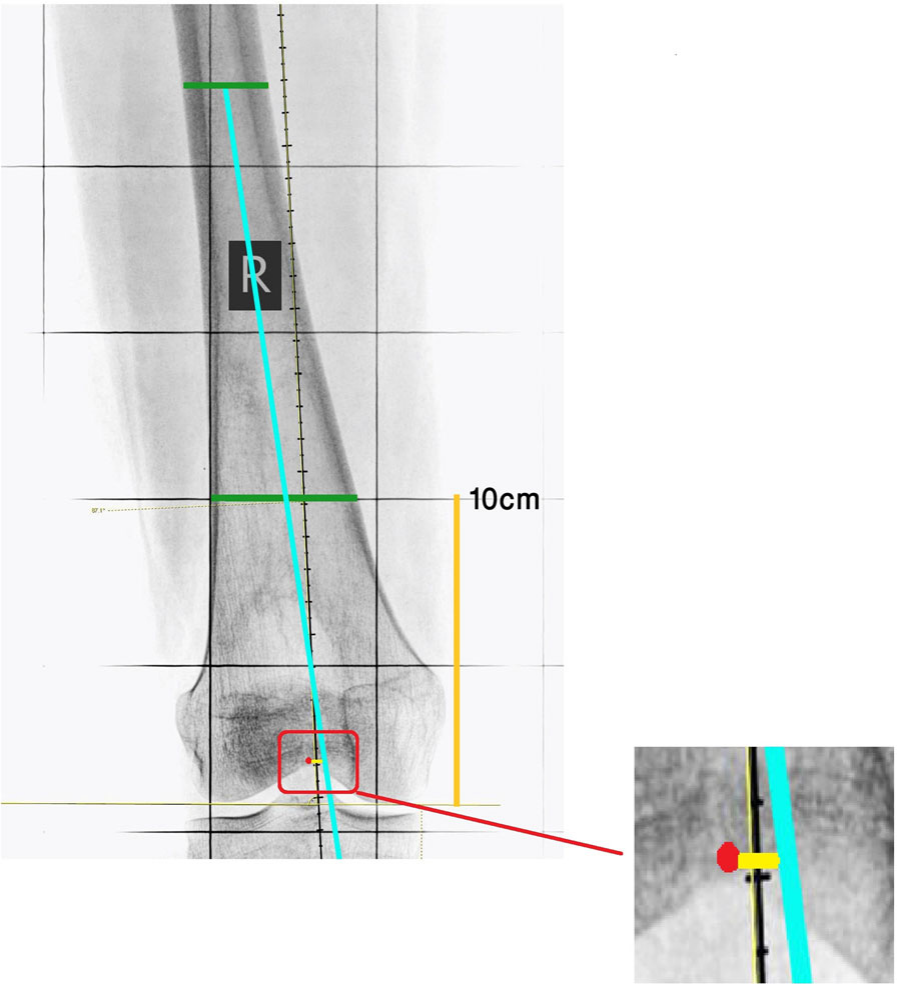
The distance (*yellow line*) between the anatomical axis of the distal half of the femur (*blue line*) and the center of intercondylar notch (*red dot*)—original image, not previously used in any article. * All X-rays are from one subject in the study. As the study was retrospective the need for consent was waived by the IRB. All patients consent to having their X-rays being used for research purposes in the hospital. All personal identifiers were removed from X-rays

In order to outline the distal anatomical femoral axis as described by Morland et al. [3], a point in the middle of the medulla 10 cm proximal to the femoral articular surface was marked. Then a line from this point to midpoint of the medulla at the mid shaft level of the femur was drawn and extended distally to cross the articular surface (Fig. 6). The horizontal distance between this line and the center of the intercondylar notch was measured as described before in millimeters (Fig. 7).

Student’s *t* test was used for statistical analysis. All data were analyzed using SPSS v19.0 (IBM SPSS, Armonk, NY, USA), and *P* values less than 0.05 were considered to be significant.

## Results

Based on the outlined inclusion and exclusion criteria, 100 patients were enrolled in this study. Forty-five cases (45%) were male and 55 (55%) were female. The average age was 34.4 ± 11.1 years (range 20 to 50 years), with 33.6 ± 10.6 in males and 35.2 ± 11.7 in females. The average distance from the proximal anatomical axis to the center of the intercondylar notch was 6.68 ± 5.23 mm (range − 10 to + 16 mm). This value was 5.83 ± 5.49 in males and 7.4 ± 4.96 mm in females with no significant differences between genders (*P* = 0.18, Table 1). The proximal anatomical axis of femur passed lateral to the center of the intercondylar notch in 12 cases (12%), medial in 84 cases (84%) and exactly central in 4 cases (4%) (Table 2). The average distance from the distal anatomical axis to the center of the intercondylar notch was 3.63 ± 2.09 mm (range, 0 to 8 mm), 3.5 ± 2.17 mm in males and 3.74 ± 2.04 mm in females with no significant differences between genders (*P* = 0.71, Table 1). The distal anatomical axis of the femur passed medially to the center of the intercondylar notch in 82 cases (82%) and exactly central in 18 cases (18%), (Table 2).

**Table 1:** The distance between projection of the proximal and distal anatomical axes of the femur to the center of the intercondylar notch

	Min (mm)	Max (mm)	Total average ± SD (mm)	Male average ± SD (mm)	Female average ± SD (mm)	*P* value
Proximal anatomical axis	− 10	16	6.68 ± 5.23	5.83 ± 5.49	7.4 ± 4.96	0.175
Distal anatomical axis	0	8	3.63 ± 2.09	3.5 ± 2.17	3.74 ± 2.04	0.712

**Table 2:** The position of the projection of the proximal and distal anatomical axes in relation to the center of the intercondylar notch

	Lateral number (%)	Center number (%)	Medial number (%)
Proximal half anatomical axis	12 (12%)	4 (4%)	84 (84%)
Distal half anatomical axis	0	18 (18%)	82 (82%)

There was a significant difference between the anatomical axis of the proximal and distal parts of the femur in reference to the center of intercondylar notch (*P* value < 0.05), supporting our hypothesis that anatomical axes of the proximal and distal halves of the femur are different in the coronal plane in the normally aligned healthy population.

## Discussion

To the authors’ best knowledge, this is the first time that the anatomical axes of the proximal and distal parts of the femur are investigated separately in the normally aligned healthy population. Also, the normal ranges for these two axes in references to the center of the intercondylar notch have not been measured previously.

Based on the distance between these 2 axes and the center of the intercondylar notch, we have showed that they are different and should be considered separately. The anatomical axis of the proximal half passes medially to the center of the intercondylar notch in most cases (84%) with an average distance of 6.68 ± 5.23 mm, whereas the anatomical axis of the distal half passes through the center of the intercondylar notch or medial to the center of the intercondylar notch in all cases (100%) with an average distance of 3.63 ± 2.09 mm.

Standing long-leg X-ray is an important method to evaluate the axial alignment of the lower extremity. The morphology of the femur and its axes are important for pre-operative assessment in femoral fracture surgeries, corrective osteotomies, and post-operative follow-ups [1, 7, 13].

There are variations in anatomical and mechanical axes of the lower extremity including the femur in normal population, considering gender and ethnicity [3, 14–16]. Several studies evaluated the knee angle variations in different countries in order to establish standards for a particular ethnicity and population [8, 12, 17, 18].

Morland et al. described different techniques to outline the anatomical axis. They emphasized that the anatomical axis of the femur never passes through the center of the knee [13], but they did not evaluate the variations in axis projections and differences between anatomical axes of the proximal and distal parts of the femur.

Reed et al. reported the distance between anatomical axis and the center of the femoral notch to be 6.6 mm, medially. Based on their findings, the entry point for femoral intramedullary guide rod in TKA should be 6.6 mm medial to the center of the femoral notch [19]. However, their study was not performed on normally aligned femurs. The differences in our findings (3.6 mm versus 6.6 mm) may be due to case selection and ethnic variation.

In our study, the average distances between proximal and distal anatomical axes of the femur to the center of the intercondylar notch were 6.68 and 3.6 mm, respectively. In terms of average distance between the proximal axis of the femur to the center of the intercondylar notch, our results are consistent with previous studies, which reported and average distance of 7 mm [20, 21].

Wangroongsub et al. (2009) evaluated the proper entry point for femoral intramedullary guides in total knee arthroplasty. Based on their results, the entry point for intramedullary guide was measured at 1.5 ± 2.01 mm medial and 12 ± 2.72 mm superior to the top of the femoral intercondylar notch, at the distal femur [22]. But in our study, we showed that according to the normal range for the anatomical axis of the distal part of the femur, the anatomical entry point would be 3.63 ± 2.09 mm medial to the center of femoral intercondylar notch.

Two major complications for trochanteric nailing used for treatment of femoral shaft fractures are varus malalignment and iatrogenic fracture [23]. The reason for these problems is straight insertion of the nail through the entry point [24, 25]. In these cases, the alignment of the lower extremity should be considered. If the nail is fitted proximally, it should follow the anatomical axis of the proximal half. For that purpose, the distance between the projection of the nail and the center of the condylar notch can be used to achieve normal alignment in coronal plane. The normal ranges for the anatomical axis of the distal part of the femur can also be helpful to find the best entry point for femoral retrograde intramedullary nailing.

Our study demonstrated that male and female subjects were similar in terms of average distances between the anatomical axes of the proximal and distal parts of the femur to the center of the intercondylar notch. While some researchers agree that the morphology of the proximal part of the femur varies in different races and between genders [26, 27], our results indicated no significant differences in measured distances between males and females.

According to Nowicki et al., computer-based and manual methods for determining lower extremity alignment from digital radiographs are not dissimilar and both provide fair to good inter-observer and intra-observer reliability [28], so we are fairly confident of our results.

One of the limitations of our study was that we did not evaluate both proximal and distal anatomical axes of the femur in the sagittal plane. Additionally, our findings were limited to a single ethnic group and limited number of patients. Furthermore, we did not check for any difference between the right and left lower extremity. Also our cases were not evenly distributed in age groups, so we could not check for the axes variability through different ages. A larger study is needed to address these limitations and to confirm our initial findings.

## Conclusions

While surgeons are aware that the anatomical axis of distal part of the femur is different from the anatomical axis of the proximal part in patients with femural deformities, we have shown that these axes are also different in the normally aligned healthy people by nature.

Furthermore the normal ranges we provided, 6.68 ± 5.23 mm to the center of the intercondylar notch for the anatomical axis of the proximal femur and 3.63 ± 2.09 mm for the anatomical axis of the distal femur, can be used as a reference point for the alignment rod entry point in surgeries such as TKA and retrograde and antegrade intramedullary femoral nailing.

Although the ideal way for determining the natural alignment of the femur remains comparing the two lower extremities, in a large number of cases it is not possible for reasons such as bilateral injuries and deformities in which there is not a normal extremity to compare to, emergency cases when there is not enough time to thoroughly plan the surgery and lack of alignment view of the other side at the time of the surgery for various reasons. In these instances as well, we propose to use the normal ranges for the anatomical axes of the proximal and distal parts of the femur, accordingly.

## Abbreviations

HKA radiographic: Hip-knee-ankle radiographic
LDFA: Lateral distal femoral angle
TKA: Total knee arthroplasty
